# A Qualitative Study on Experiences and Perspectives of Members of a Dutch Medical Research Ethics Committee

**DOI:** 10.1007/s10730-019-09394-4

**Published:** 2019-12-27

**Authors:** Rien M. J. P. A. Janssens, Wieke E. van der Borg, Maartje Ridder, Mariëlle Diepeveen, Benjamin Drukarch, Guy A. M. Widdershoven

**Affiliations:** 1 Department of Medical Humanities, Amsterdam UMC, Location VUmc, PO Box 7507, 1007 MB Amsterdam, The Netherlands; 2 Department of Anatomy and Neurosciences, Amsterdam UMC, Location VUmc, PO Box 7507, 1007 MB Amsterdam, The Netherlands

**Keywords:** Ethics Committee, Research ethics, Human subjects, Responsibility

## Abstract

The aim of this research was to gain insight into the experiences and perspectives of individual members of a Medical Research Ethics Committee (MREC) regarding their individual roles and possible tensions within and between these roles. We conducted a qualitative interview study among members of a large MREC, supplemented by a focus group meeting. Respondents distinguish five roles: protector, facilitator, educator, advisor and assessor. Central to the role of protector is securing valid informed consent and a proper risk-benefit analysis. The role of facilitator implies that respondents want to think along with and assist researchers in order to help medical science progress. As educators, the respondents want to raise ethical and methodological awareness of researchers. The role of advisor implies that respondents bring in their own expertise. The role of assessor points to contributing to the overall evaluation of the research proposal. Various tensions were identified within and between roles. Within the role of protector, a tension is experienced between paternalism and autonomy. Between the role of protector and facilitator tensions occur when the value of a study is questioned while risks and burdens for the subjects are negligible. Within the role of assessor, a tension is felt between the implicit nature of judgments and the need for more explicit formulations. Awareness of various roles and responsibilities may prevent one-sided views on MREC work, not only by members themselves, but also by researchers. Tensions within and between the roles require reflection by MREC members.

## Introduction

### Background

Currently, in the Netherlands there are 20 Medical Research Ethics Committees (MRECs), which independently review medical scientific research protocols involving human subjects. While Hospital Ethics Committees typically advise professionals and board members in hospitals on ethical cases, dilemmas and policies, MRECs in the Netherlands are independent administrative bodies invested with the jurisdiction to approve or disapprove of medical scientific studies involving human subjects. In line with Dutch law, MRECs foster valid informed consent and assess whether the risks and burdens for the subjects are proportionate to the expected benefits for the subjects and/or future patient populations. This latter assessment illustrates that MRECs, next to protecting participating subjects, also have other responsibilities that relate to the quality and progress of medical science (Hunter 2007; Garrard and Dawson 2005; Guillemin et al. 2012). This raises the question which roles MREC members see for themselves and which responsibilities they adopt. Protecting the interests of subjects on the one hand and facilitating the researcher on the other, can bring along moral tensions (de Jong et al. 2012, 2013; Zon 2012). Empirical studies in the USA revealed how MREC members face dilemmas and experience tensions in their relationship with researchers (Klitzman 2011a, 2012, 2013). These studies, however, focussed mainly on the operation of the committee as a whole, and provided only scant information concerning the experience(s) and views of individual committee members on their roles and responsibilities.

To gain in-depth insight into the perspectives of MREC members on their individual roles and responsibilities and on possible moral tensions within and between these roles, we conducted a qualitative study among members of a large Dutch MREC attached to a University Medical Center (UMC). The specific research questions addressed were: *What is the perspective of MREC members on their roles and responsibilities? Which tensions are experienced in and between these roles and how are these dealt with?*

## Method

### Recruitment and Design

The study took place between February 2016 and November 2017. The Executive Board of the MREC (chairman, vice-chairman and secretaries) provided written information about the study to all MREC members. Of those members who indicated interest to participate, twelve were eventually interviewed taking into account variety among participants with regard to sex and professional disciplines. In order to protect privacy and avoid identifiability we will not specify the background of the participants.

A qualitative research design was used (Guba and Lincoln 1989; Abma and Widdershoven 2006). Semi-structured interviews were conducted by two researchers [MD, WB], who used a topic list (see Appendix). The interviews took place at the workplace of the participants, and lasted 30 to 60 minutes. All interviews were recorded (audio) and transcribed ad verbatim. Saturation was reached after ten interviews; the last two interviews did not yield any new information (Loiselle et al. 2007; Mays 2000; Barbour 2001). Summaries of interview transcripts were shared with the respondents (member check) and their suggestions were adopted in the final report.

After completion and analysis of the interviews, a focus group was organised. The purpose of this meeting was to obtain individual and group feedback and clarification and/or elaboration on topics selected by the research team. The meeting (duration 1.5 hours) was attended by 21 participants (12 MREC members, 4 secretaries and 5 staff employees). One of us [GW] chaired the discussion on the basis of a discussion guide. Other members of the research team (RJ, WB, BD, MR) were present and made notes. The report of the meeting was handed out to all participants (extra member check). No suggestions for amendments were received.

### Analysis

The analysis took place during the data collection process, and guided further data collection (iterative process). The data was analysed thematically on content. This means that the data were read closely while meaningful fragments were coded (open coding), categorized (selective coding), and then clustered (axial coding) (Boeije 2010). Themes and issues from the perspectives of the various stakeholders were gathered (Guba and Lincoln 1989; Abma and Widdershoven 2006; Stake 2004). The analysis program MaxQda 12 was used. To increase reliability, all transcripts and reports were coded and analysed by at least two researchers, independent of one another. Analyses, themes and categories were repeatedly discussed in the research team until consensus was reached.

### Ethical Considerations

Prior to the start of the study, the MREC issued an official declaration, stating that according to Dutch law the study is not in need of review by a MREC.

All participants were informed both orally and in writing about study procedures, confidentiality, and privacy procedures. All participants signed an informed consent form and gave permission for recording the interview and using the interview data. The research data were stored in a secured project file. Access to the study data was limited to members of the research team. Audio recordings of interviews were destroyed after transcripts were made.

## Results

### Five Roles

The respondents distinguished five roles: protector, facilitator, educator, advisor and assessor. Each of these roles brings along specific tasks and responsibilities; tensions are reported, both within and between roles. Below, the tasks and responsibilities are described for each role as well as the tensions.

### Protector

According to all respondents (and in line with the wording and intentions of the pertaining Dutch laws and regulations), the central responsibility of the MREC is to protect the rights, security and well-being of subjects who participate in medical scientific research.

A first task associated with the role of protector is to assess and minimize risks and burdens for the subjects. This applies to subjects who are exposed to an experimental intervention, but also to subjects from whom the intervention or even treatment are to be withheld.

“If, for example, in the control group, treatment is withheld for a certain period, yes then you should have good reasons for doing that. Safety assurance is needed, in order to guarantee that the patient does not get worse during that period.”

To be able to make an assessment of risks and burdens, researchers are instructed to be very explicit about the risks and burdens in the protocol. Respondents try to empathize with the subjects.

“You often imagine what is actually done to the subject. And what impact this can have.”

A second task in the context of protection is to ensure valid informed consent, implying that the subject is enabled to voluntarily decide to participate, based on accurate, comprehensible, and complete information. Several respondents emphasized the importance of scrutinizing the quality of the patient information leaflet. Addressing the subject in an empathic way was considered important.

A limitation in assessing burdens and risks is that the subject’s actual experience of burdens is difficult to determine.

“I think that we, and certainly the clinicians, have a certain bias about what the experienced burden of a treatment is for patients.”

A limitation to the assessment of information is that the way in which oral information is provided to the subject is invisible for MREC members. This is problematic because the way in which the subject is approached may affect the voluntary character of the patient’s consent.

“But there is often discomfort in me. […] Is our interpretation of the informed consent equal to the informed consent as it is experienced by this potential patient group?”

A tension within the role of protector regards the dilemma between paternalism and autonomy. On the one hand, subjects need to be protected from burdensome research. On the other hand, subjects need space to decide for themselves. In the MREC, the tension between the two perspectives is not made explicit. As a consequence, according to a respondent, a reference to the subject’s own responsibility, emphasizing autonomy, may hinder the ethical discussion about the risks and burdens, which is important, especially when vulnerable groups are involved.

“Can you still do that to someone in the final phase of life? […] The discussion about this quickly ends and goes like: ‘yes, but people can decide for themselves’.”

### Facilitator

A second role that was identified is being a facilitator of research. The respondents experience the responsibility to help medical science progress.

“You want to help science progress, don’t you? […] Not take the position of ‘nothing goes’. […] after all, we are an academic clinic.”

Facilitating research implies creating room for researchers and thinking along with them in creative ways. Facilitating research also serves the interests of the organization. These interests were also experienced as a responsibility.

“Political interests are also at stake. The MREC wants to continue its existence, just like the university does.”

There is a tension between the role of facilitator and the role of protector, as protection of the subject can compromise members in facilitating research. A respondent expressed the way the MREC deals with this tension, by advising the researcher to change the design if benefits do not outweigh risks and burdens.

“In this way it does not work, but try to solve it in a different way because as […] you are going to do it now, it can turn out bad for the subjects.”

Dilemmas are experienced especially if research has questionable scientific value, but entails very little risks and burdens. If such research is approved of, it may give false hope to subjects.

Some respondents experience tensions when reviewing protocols that have already received funding after peer review. If MRECs require substantial changes, renewed approval from the funding agency may be required. Also, especially with regard to large, international studies, there is virtually no room for suggestions for improvement or other changes/amendments.

“It is difficult to influence a study, as a single committee. […] A big international research project in which the Netherlands provides 10 percent of patients that already started in other countries, yes then it is difficult.”

### Educator

The role of educator is concerned first with making researchers aware of the burden and risks to which the subject is exposed, and second with helping to improve (present and future) scientific quality of research. This is done by stimulating researchers to reflect, based on suggestions and questions from the MREC.

“We encourage, by asking questions, the researcher to think about whether or not that burden can be minimized. Or, what’s more even, whether such research should be designed in another way.”

This not only concerns a learning effect on the short term, but also on the longer term. Critical comments on a protocol can educate the researcher and help him/her submit better future research proposals. Specific support is also offered by inviting the researcher to contact individual MREC members for help. Some participants mentioned that they also talk with researchers outside the meeting, on the researcher’s request or on their own initiative.

“I suggested to the researcher, well I’ll sit down with you and then I can explain what the underlying idea of all those questions is.”

There are tensions between the roles of educator and facilitator. Researchers are not always interested in learning about methodological and ethical issues. Instead, they want to move forward with their research.

“So there are researchers with whom you occasionally have a bit of an annoying conversation. […] And then I always try to explain, well, that if the protocol is not methodologically sound […] yes then the burden for the patients is not ethical anymore.”

The educator role is often limited to contact with individual researchers. Some respondents favour an educational responsibility of the MREC in the direction of whole research groups or departments.

### Advisor

The role of advisor concerns bringing in knowledge and expertise of individual members during the evaluation process. In the context of this role, members assess whether the protocol is complete and accurate regarding the area of their own expertise. For example, literature references are checked to see if results from earlier studies agree with the information provided by the investigator(s).

Sometimes background information about the context of the research is also given by individual members, for instance regarding the patent terms of standard drugs.

“I say ‘yes the patent is finished soon guys’. So do a small innovation and then they can again ask the maximum prize next year.”

Since input as advisor is often based primarily on their highly specialized expertise, individual MREC members do not always review all parts of a protocol. As a result, their individual role is sometimes described as being modest.

“Yes, my role is very limited in the sense that when […] things come up in my area, that I am then supposed to talk about it.”

Moreover, respondents without a medical background in general value their individual contribution as limited and indicate that physicians and pharmacologists have a more important influence as advisor. These experiences can be seen as tensions within this role.

### Assessor

Assessment and judgement are considered as crucial responsibilities. The respondents use their individual expertise in assessing the protocol, yet also experience a responsibility for the quality of judgment of the MREC as a whole. They indicated that they are dependent on one another and strive for jointly formulating a judgment, using a process of consensus formation.

“Everyone is there with his own field of expertise. One cannot judge such a study on one’s own, that is just a combination of all ideas. And there you need one another.”

Respondents see it as their task and responsibility to assess similar protocols in similar ways. Although not mentioned by all respondents, consistency in judgement(s) is considered important, to prevent ad hoc decisions and lack of clarity for researchers.

“I try to evaluate every protocol in the same way.”

The role of assessor presupposes independence. This can lead to tensions when MREC members assess a protocol on which they were already asked for advice by the researchers when writing the protocol.

“I do a lot of research within the hospital. I am often asked for advice regarding a protocol on [specific field of expertise] and then there are protocols that come back to the MREC. So there is a little bit, yes, a conflict.”

It also happens that conflicts of interest are at stake when protocols from group or departmental colleagues are discussed. It is considered important that these interests are reported.

A tension is seen regarding the relationship between technical and ethical aspects of the assessment. A relatively large amount of attention is invested into the scientific aspects of the protocol. That is partly due to the large number of researchers in the committee. When the discussion primarily focuses on scientific quality, this can, according to a number of respondents, detract from the ethical discussion. In this sense, a tension is felt between the roles of assessor and protector.

“I think the statistical considerations and also the medical considerations are discussed very well, as far as I can judge. But sometimes I miss the ethical discussion. […]The discussion just runs very fast. I think, yes, I would need more ethical discussion about things.”

Judgement and consensus formation are often implicit. This may create a tension within the role of assessor, since criteria are not clearly formulated. It can also result in a tension between the role of facilitator and that of educator, as criteria are not explained to the researcher. Some respondents argue that consensus formation should be more explicit.

“And that very explicit weighing of benefit versus burden either happens implicitly and is therefore invisible, or does not happen at all. My opinion on this field of tension is that we should make consensus formation more explicit and thereby increase quality.”

The implicit way in which the protocols are assessed and judged implies that a protocol is hardly ever explicitly rejected. The outcome of the assessment process is a positive review, or additional questions are asked, which the researcher must satisfactorily answer. This can lead to a tension between the role of facilitator and the role of assessor.

“The strength of our MREC is that a compromise is always sought. […] How can we still enable the researcher to do this research with adjustments, I really like that. But it is also a weakness.[…] Actually, I have […] experienced only once that something was rejected.”

In contrast to explicit rejection, “implicit rejection” sometimes happens, for instance when a researcher is overloaded with questions and comments, making the probability of a (thoroughly) revised version very low.

“Sometimes we give so many comments and so much has to be changed, that the researcher thinks ‘I withdraw the protocol’. Of course that does happen.”

## Discussion

As far as we know, this is the first qualitative study into the roles and responsibilities identified by individual members of a MREC. Previous research focused on responsibilities of MRECs in their entirety (de Jong et al. 2012; Klitzman 2011a, b; Angell et al. 2008). Our analysis identified five roles: protector, facilitator, educator, advisor and assessor. The respondents indicate that the protection of the research subject is their first responsibility, which is in accordance with national and international declarations and guidelines such as the Helsinki Declaration and the CIOMS guidelines. According to the respondents, central to this role is the informed consent demand and the risk–benefit analysis. The role of facilitator indicates that respondents value the progress of medical science and want to assist researchers to meet ethical requirements so that the study can proceed. As an educator, the respondents want to raise awareness of researchers, in particular with regard to the burden for the subjects and the scientific quality of the study (Klitzman 2013; Angell et al. 2008). In the role of advisor, MREC members bring in their individual expertise during the deliberation of the committee. In the role of assessor, each MREC member contributes to judging the moral acceptability of the protocol, which is regarded as a joint endeavour.The distinction between the roles described above is analytical. In practice, roles may overlap, MREC members may experience different roles at the same time and, perhaps, depending on the complexity of the protocol, the importance attached to roles may vary. Educational activities may also have a protective value. Assessment of protocols will often if not always also amount to protection of research subjects and this may at times go hand in hand with facilitation of research. Which roles are dominant in the assessment of protocols may be subject to “hidden dynamics” among the multidisciplinary membership. In this respect, the role of the (vice)chair of the MREC and the space (s)he gives to the various roles could be of importance. Further qualitative and observational research is necessary to shed light on these hidden dynamics (see, for example, Rangel 2009). Earlier research in an MREC concluded that in the review of studies, two “repertoires” can be distinguished: a repertoire of rules, related to an approve/disapprove approach, and a repertoire of production, related to an “improve” approach (de Jong et al. 2012). The repertoire of rules focuses on the risk–benefit analysis and informed consent, while in the repertoire of production the progress of medical science and cooperation with the researcher is paramount. In our analysis of the five roles, the two repertoires are also visible. Yet, more clearly than in the names of the two repertoires, ethical aspects of the work of members of the MREC stand out. In the context of rules and regulations, MREC members show concern about values, such as patients’ well-being. In the context of contributing to production, MREC members not only focus on helping the researcher to get a favourable review, but also to improve protocols in ethical and scientific terms. Respondents feel shared responsibility with the researchers regarding the moral and scientific quality of medical scientific research with human subjects.

Our study revealed tensions within and between roles. Within the role of protector, a tension is experienced between paternalism and autonomy (Garrard and Dawson 2005; Edwards et al. 2004; Savulescu 1998). Subjects need protection, but also have a right to decide for themselves whether or not to take part in a study. Between the role of protector and facilitator a tension occurs when methodologically unsound research is considered unethical since subjects must be protected against research without or with very little value. This can raise objections from researchers, especially if their study already received funding or is part of a large multicentre study. In the role of assessor, a tension can be seen between the implicit nature of considerations and judgments and the need felt by some respondents for more explicit formulations. Implicit assessment is part of the process of facilitating the researcher, but it may result in lack of clarity, both in the committee and for the researchers. Tensions such as those described here show that the work of members of the MREC is guided by different values that can come into conflict with one another. Respondents in general do not experience the tensions as problematic. Yet, some members of the committee indicate that more explicit balancing of values may clarify judgements, both for the committee itself and for the researchers. In clinical situations, teams can be assisted in making explicit values and finding solutions in case of dilemmas, by methods for clinical ethics support, such as moral case deliberation (de Haan et al. 2018; Abma et al. 2009; Weidema et al. 2011, 2013). This approach might also be useful to foster reflection in MRECs concerning tensions between roles and responsibilities.

### Strength/Weakness

The results of this research are based on individual interviews and a focus group discussion with MREC members. The combination of individual interviews and a focus group led to more in-depth insight. Representatives of all disciplines were interviewed, and in the focus group a large number of members of the MREC participated.

This study was conducted in one MREC only. Different roles and responsibilities and different tensions may be seen in other MRECs, for instance MRECs that are not attached to UMCs or MRECs that typically assess studies from researchers who are unknown to the members. Given the exploratory nature of the study, further research in other MRECs is needed in order to validate our findings. As mentioned above, complexities regarding decision making processes in groups and hidden dynamics among multidisciplinary members may have come insufficiently to the surface and perhaps other designs, such as long term observations, can have surplus value in addressing these issues.

### Recommendations

Awareness of various roles and responsibilities may prevent one-sided views on MREC work, not only by members, but also by researchers. In times of digitalization and economization of MRECs, space should stay available for MREC members to reflect together, on themselves as individual members, and on the specific MREC in its entirety. One way of raising awareness of roles and responsibilities among MREC members may be to stimulate visits of other MRECs, following different procedures and having different dynamics. Members should be given time, and financial resources, to further develop themselves, raise awareness, and learn from other members, as well as from other MRECs. This may even reveal new roles and responsibilities (and tensions between them), that have remained unconscious so far. In case of dilemmas resulting from conflicting roles and responsibilities, reflection, for instance in the form of Moral Case Deliberation, may support decision making. Follow-up research among members of other RECs is recommended in order to confirm and refine the results of this study.

## Appendix—topic list interviews

Aim: to identify different roles, responsibilities, perspectives and interests of REC members.

Information about interview

- Introduce oneself
- Explain the structure of the interview
- Explain research
- Composition of research team
- Duration of the interview
- Purpose of the interview
- Request permission for use of the interview
- Anonymity
- Member check possible?
- Obtaining oral and written informed consent

Background respondent

- Profession (general)
- Duration of membership REC
- Other experience with REC

Questions relating to research question

1. Role and responsibility REC member
  - motivation to become a REC member
  - description and interpretation of one’s own role
  - responsibility for one’s own role
  - interests in one’s own role
  - recognition of other roles
  - relation of one’s own role to the role of others
  - recognition of roles as protector and stakeholder
2. Role and responsibility REC
  - description of the role of the REC (within science and hospital)
  - elaboration of the role of the REC in practice
  - responsibility of the REC
  - different roles of the REC as a whole
  - interests of the REC
3. Mentioning this part of the interview will lead to identification of the REC. None of the issues described in the manuscript relate to this part
4. Dilemmas/tensions
  - tensions in daily practice (roles, interests, responsibilities)
  - responsibility of REC member vs. REC as a whole
  - interests of REC member compared to REC as a whole
  - addressing tensions/dilemmas
  - how to deal with tensions and dilemmas
  - influence of tensions on process of assessment and dynamics (atmosphere/quality)

**NB** Ask as much as possible for concrete examples.
